# Causes of Infection after Earthquake, China, 2008

**DOI:** 10.3201/eid1606.091523

**Published:** 2010-06

**Authors:** Yue Wang, Peng Hao, Bo Lu, Hua Yu, Wenfang Huang, Hongliang Hou, Kerong Dai

**Affiliations:** Sichuan Academy of Medical Sciences and Sichuan Provincial People’s Hospital, Chengdu, Sichuan, People’s Republic of China (Y. Wang, P. Hao, B. Lu, H. Yu, W. Huang); Ninth People’s Hospital, Shanghai, People’s Republic of China (H. Hou, K. Dai); Shanghai Jiaotong University School of Medicine, Shanghai (H. Hou, K. Dai)

**Keywords:** Earthquake, infection, etiology, gas gangrene, drug resistance, bacteria, fungi, China, dispatch, expedited

## Abstract

To determine which organisms most commonly cause infection after natural disasters, we cultured specimens from injured earthquake survivors in Wenchuan, China, 2008. Of 123 cultures, 46 (59%) grew only 1 type of pathogenic bacteria. Smear was more effective than culture for early diagnosis of gas gangrene. Early diagnosis and treatment of wounds are crucial.

On May 12, 2008, an 8.0-magnitude earthquake occurred in Wenchuan, People’s Republic of China. For many survivors who were hospitalized, wound or other generalized infections developed. To learn more about relevant factors and therapeutic solutions for infections in earthquake survivors, we analyzed the characteristics and antimicrobial drug susceptibility of organisms cultured from wounds, blood, and sputum of persons admitted to Sichuan Provincial People’s Hospital after the earthquake. This hospital, 1 of the biggest public hospitals in the earthquake zone, admitted 2,131 survivors, including 856 patients who had been transferred from other hospitals after preliminary treatment; 2,105 recovered and were discharged by July 31, 2008. Thus, this hospital’s patients may be representative of all survivors of the Wenchuan earthquake.

## The Study

Earthquake sequelae were as follows: among survivors, 1,970 (92.4%) had limb or spinal trauma, 108 had head injuries, and 40 had thoracic injuries. Among the 78 patients in whom testing of wound secretions, sputum, or blood samples yielded positive results, 65 (83.3%) had fractures, including 38 with open limb fractures and 29 with multiple fractures; 35 (44.9%) had crush injuries; 15 (19.2%) had renal dysfunction; 30 (38.5%) had pulmonary injury or recurrent or aggravation of existing pulmonary disease; and 19 (24.4%) had gas gangrene. We included in our study those patients who had been injured in the earthquake and admitted to Sichuan Provincial People’s Hospital from May 12 through July 31, 2008, and who had any of the following conditions: increased wound secretion after debridement, indicating possible infection; continuous fever >38.5°C and pulmonary signs and symptoms, indicating possible pulmonary infection; continuous fever that could not be explained by wound or specific organ infection or fever with suspicion of bacteremia; and clinical signs indicating special infections such as gas gangrene.

To identify bacteria and fungi, we cultured 571 specimens of wound secretions, sputum, or blood from 123 patients (74 male and 49 female); results were positive for 78 patients. Of these 78 patients, 46 were male and 32 were female, average age was 49 years (range 9–95 years), and 13 were <20 and 31 were >60 years of age. From these 78 patients, 19 had specimens collected from 1 location, 28 from 2 locations, 14 from 3 locations, and 17 from >4 locations. We also conducted anaerobic culturing of wounds with suspected gas gangrene.

An average of 3 cultures were performed for each patient. All cultures for patients who had multiple-collection or multiple-location specimens that grew consistent bacterial strains were considered as 1 culture. Bacteriologic and antimicrobial drug susceptibility testing was performed by using a VITEK-32 automatic microbial analyzer or an ATB automatic microbial analyzer (both from bioMérieux, Durham, NC, USA). Drugs tested were cefepime, ceftazidime, aztreonam, ciprofloxacin, gentamicin, imipenem, piperacillin/tazobactam, sulfamethoxazole/trimethoprim, and amikacin.

Of the 571 specimens, positive culture results were obtained from 169 (59.5%) of 284 wound secretions, 120 (61.2%) of 196 sputum samples, and 22 (24.2%) of 91 blood samples (Table 1). Culture results indicated that 46 (59.0%) patients were infected with 1 type of pathogenic bacteria and 31 were infected with >1 type (21 [26.9%] with 2 types, 7 [9.0%] with 3 types, and 4 [5.1%] with >4 types). Of those infected with >1 type of bacteria, 15 (46.9%) were >60 years of age. The predominant bacteria causing infection were *Acinetobacter baumannii*, *Escherichia coli*, and *Staphylococcus aureus*. Antimicrobial drug susceptibility testing results for the 5 most common among the 180 strains of isolated gram-negative bacilli are shown in Table 2.

**Table 1:** Pathogenic bacteria isolated from persons injured during earthquake, Wenchuan, China, 2008

Source	Bacteria, no. (%)*											
	*A.b.*	*E.c.*	*S.a.*	*K.p.*	Enteric bacilli	*P.a.*	*C.a.*	CN staph	*S.m.*	*S.s.*	Others	Total
Wound	42 (24.9)	26 (15.4)	20 (11.8)	16 (9.5)	16 (9.5)	13 (7.7)	0	0	0	0	36 (21.3)	169
Sputum	29 (24.2)	6 (5.0)	12 (10.0)	6 (5.0)	12 (10.0)	5 (4.2)	18 (15.0)	6 (5.0)	0	0	26 (21.6)	120
Blood	3 (13.6)	4 (18.2)	6 (27.3)	0	2 (9.1)	0	1 (4.5)	0	2 (9.1)	2 (9.1)	2 (9.1)	22

*A.b., Acinetobacter baumanii; *E.c., Escherichia coli; S.a., Staphylococcus aureus; K.p., Klebsiella pneumoniae; P.a., Pseudomonas aeruginosa; C.a., Candida albicans;* CN staph, coagulase-negative staphylococci*; S.m., Serratia marcescens; S.s.,Staphylococcus saprophyticus.*

**Table 2:** Antimicrobial susceptibility of 180 strains of gram-negative bacilli isolated from persons injured during earthquake, Wenchuan, China, 2008

Organism	No.	No. (%) specimens susceptible, by antimicrobial drug*								
		AMK	AZT	CAZ	CIP	FEP	GM	IMP	SMZ	TZP
*A. baumannii*	74	16 (21.6)	0	5 (6.8)	7 (9.5)	15 (20.1)	0	26 (35.1)	10 (13.5)	12 (16.2)
*Escherichia coli*	36	36 (100)	13 (36.1)	13 (36.1)	0	11 (30.6)	10 (27.8)	35 (97.2)	11 (30.6)	28 (77.8)
*Enteric bacilli*	30	15 (50.0)	10 (33.3)	10 (33.3)	8 (26.7)	13 (43.3)	13 (43.3	30 (100)	8 (26.7)	10 (33.3
*Klebsiella pneumoniae*	22	20 (90.9)	4 (18.2)	7 (31.8)	0	7 (31.8)	4 (18.2)	22 (100)	4 (18.2)	7 (31.8)
*P. aeruginosa*	18	18 (100)	4 (22.2)	11 (61.1)	12 (66.7)	11 (61.1)	15 (83.3)	17 (94.4)	0	15 (83.3)
Total	180	105 (58.3)	31 (17.2)	46 (25.6)	27 (15.0)	57 (31.7)	42 (23.3)	130 (72.2)	33 (18.3)	72 (40.0)

*Semisusceptible results were considered resistant. AMK, amikacin; AZT, aztreonam; CAZ, ceftazidime; CIP, ciprofloxacin; FEP, cefepime; GM, gentamicin; IMP, imipenem; SMZ,sulfamethoxazole/trimethoprim; TZP, tazobactam/piperacillin; *A. baumannii, Acinetobacter baumannii; P. aeruginosa, Pseudomonas aeruginosa.*

To detect suspected cases of gas gangrene, we harvested 53 smear specimens, of which 22 produced gram-positive thick bacilli (18 with capsules and 4 without). Further culturing after a period of isolation and treatment of the patients showed the pathogen for 4 patients to be a non–gas-producing bacillus. Of these 22 patients, 3 did not have gas gangrene, but the remaining 19 did, confirmed by culture and clinical signs (accuracy rate 86.4%). Culture results for another 10 patients showed other bacteria (intermediate-size gram-positive bacilli).

Risk for nosocomial infection might have been increased because many patients had been treated with antimicrobial drugs at other hospitals before being transferred to Sichuan Provincial People’s Hospital. Instead of genotype testing (because resources were limited), we used the resistance spectra of bacteria to differentiate nosocomial from primary infection. *A. baumannii* and *E. coli* are gram-negative bacilli that exist widely in the environment, mainly in water and soil, the main site for development of antimicrobial drug resistance (*1*,*2*). Because *A. baumannii* were strongly resistant and because resistance spectra for several strains of *A. baumannii* were nearly identical, we concluded that any infections with this pathogen were probably obtained during hospitalization. In contrast, because the resistance spectra of *E. coli* and enteric bacilli varied substantially, we concluded that infections with these bacteria were most likely obtained before hospitalization. Average susceptibility of several gram-negative bacilli (except *A. baumannii*) to imipenem was >90%; susceptibility to aminoglycosides, cefepime, and ceftazidime was also high.

Among the 49 strains of staphylococcal bacteria isolated (38 strains of *S. aureus* and 11 strains of other coagulase-negative staphylococci), 39 (79.6%) strains were methicillin resistant. Susceptibility test results (no. susceptible/% susceptibility) were as follows: 49/100% for vancomycin and linezolid; 47/95.9% for quinupristin/dalfopristin; 36/73.5% for sulfamethoxazole/trimethoprim; 25/51.0% for chloramphenicol and rifampicin; 14/28.6% for clindamycin; 13/26.5 for gentamicin; 11/22.4% for erythromycin, moxifloxacin, and ampicillin/sulbactam sodium; and 10/20.4% for cefazolin and gatifloxacin.

For fungi, 19 strains were detected. All were 100% susceptible to amphotericin B, 5-fluorouracil, fluconazole, voriconazole, and itraconazole.

## Conclusions

More attention should be paid to early diagnosis and treatment of multiple infections and special infections in survivors of natural disasters. One such infection, gas gangrene, is an acute, severe, life-threatening disease for which early diagnosis is critical. For diagnosis of gas gangrene, smear examination of wound secretions is a valuable diagnostic tool and is more effective than culture for early diagnosis. No nosocomial spread of gas gangrene occurred during treatment.
